# Machine Learning Approach to Identifying Empathy Using the Vocals of Mental Health Helpline Counselors: Algorithm Development and Validation

**DOI:** 10.2196/67835

**Published:** 2025-04-16

**Authors:** Ruvini Sanjeewa, Ravi Iyer, Pragalathan Apputhurai, Nilmini Wickramasinghe, Denny Meyer

**Affiliations:** 1 School of Health Sciences, Swinburne University of Technology, Hawthorn, PO Box 218, John Street, Melbourne, 3122, Australia, 61 422587030; 2 School of Computing, Engineering & Mathematical Sciences, La Trobe University, Melbourne, Australia

**Keywords:** vocal features, voice characteristics, empathy, mental health care, crisis helpline service

## Abstract

**Background:**

This research study aimed to detect the vocal features immersed in empathic counselor speech using samples of calls to a mental health helpline service.

**Objective:**

This study aimed to produce an algorithm for the identification of empathy from these features, which could act as a training guide for counselors and conversational agents who need to transmit empathy in their vocals.

**Methods:**

Two annotators with a psychology background and English heritage provided empathy ratings for 57 calls involving female counselors, as well as multiple short call segments within each of these calls. These ratings were found to be well-correlated between the 2 raters in a sample of 6 common calls. Using vocal feature extraction from call segments and statistical variable selection methods, such as L1 penalized LASSO (Least Absolute Shrinkage and Selection Operator) and forward selection, a total of 14 significant vocal features were associated with empathic speech. Generalized additive mixed models (GAMM), binary logistics regression with splines, and random forest models were used to obtain an algorithm that differentiated between high- and low-empathy call segments.

**Results:**

The binary logistics regression model reported higher predictive accuracies of empathy (area under the curve [AUC]=0.617, 95% CI 0.613‐0.622) compared to the GAMM (AUC=0.605, 95% CI 0.601‐0.609) and the random forest model (AUC=0.600, 95% CI 0.595‐0.604). This difference was statistically significant, as evidenced by the nonoverlapping 95% CIs obtained for AUC. The DeLong test further validated these results, showing a significant difference in the binary logistic model compared to the random forest (D=6.443, *df*=186283, *P*<.001) and GAMM (Z=5.846, *P*<.001). These findings confirm that the binary logistic regression model outperforms the other 2 models concerning predictive accuracy for empathy classification.

**Conclusions:**

This study suggests that the identification of empathy from vocal features alone is challenging, and further research involving multimodal models (eg, models incorporating facial expression, words used, and vocal features) are encouraged for detecting empathy in the future. This study has several limitations, including a relatively small sample of calls and only 2 empathy raters. Future research should focus on accommodating multiple raters with varied backgrounds to explore these effects on perceptions of empathy. Additionally, considering counselor vocals from larger, more heterogeneous populations, including mixed-gender samples, will allow an exploration of the factors influencing the level of empathy projected in counselor voices more generally.

## Introduction

Empathy is defined as experiencing the emotions (emotional empathy) and cognitions (cognitive empathy) of others and responding to them appropriately [1]. Empathy is especially important for patient care, where the lived experience of the patient must be understood by responding health care professionals, while also conveying this understanding in conjunction with a desire to help the patient [2]. The effectiveness of physician empathy has been shown to improve patient satisfaction and commitment to recovery while reducing anxiety and distress levels, leading to better clinical results [3]. Furthermore, empathic behavior by mental health (MH) care providers reduces their own risk of burnout [4].

Telephone helpline services offer an effective means of supporting those who need immediate MH care [5]. The demand for such services has increased dramatically since the outbreak of the COVID-19 pandemic [6], increasing the expectations of counseling staff to provide support for people with complex MH concerns [7]. As a basic counseling skill, empathy is key to successful engagement with patients in the context of complex psychosocial needs.

Besides emotional and cognitive empathy in understanding the status of a patient, contextual awareness is equally important for therapeutic engagement [8]. This means that empathic responses need to be contextually appropriate by considering environmental cues, culture, demographic factors, and the specific circumstances of the patient to understand the broader context of their MH status [9,10]. This allows counselors to tailor responses, based on context, to engage in effective communication with distressed patients, thereby delivering better outcomes [3].

Verbal cues and tone of voice are crucial when communicating empathy [11]. For example, reduced speech rate and lower pitch are perceived as more empathic by patients when receiving bad news from health care providers in an oncology setting [12] and while actively listening to telephone callers, nurses have been found to express empathy through their choice of words, voice and intonation, projection of compassion and warmth, as well as “tuning in” to the caller’s story and identifying with the caller’s emotions [13].

Unfortunately, the global demand for MH support is not being met by the existing workforce [14]. This service gap is leading to growing interest in alternative digital technological solutions. Technological innovations, such as the design of conversational agents, have demonstrated potential in facilitating effective and immediate patient care. However, to optimize upon end user acceptance, conversational agents need to display empathy [15,16]. However, we have yet to identify the precise vocal features most associated with an empathic human response, and it is also not known if it is possible to categorize empathy levels using such vocal features. Thus, the aim of this paper is to (1) identify the vocal features significantly associated with empathy in a large collection of telephone helpline counseling call recordings; and to (2) evaluate the accuracy of a machine learning algorithm to correctly designate short segments of each recording to categories of low and high empathy.

This study has been reported in accordance with the TRIPOD+AI (Transparent Reporting of a Multivariable Prediction Model for Individual Prognosis or Diagnosis+Artificial Intelligence) checklist as shown in Checklist 1.

## Methods

### Data Collection

Recordings of telephone helpline calls (n=57) were obtained from On The Line, Australia, a suicide helpline counseling service. Participants were counselors for a Suicide Call-Back Service (a national helpline service coordinated by On The Line, Australia). Calls were randomly sampled from July 1, 2019, to June 30, 2021, stratified by organizationally determined suicide risk level (high or low). The level of suicide risk of each caller had been previously assessed by counselors using the Columbia Suicide Severity Rating Scale (C-SSRS) to differentiate between calls featuring high suicide risk (with C-SSRS ratings of 6‐7) and calls with low risk of suicide (with C-SSRS ratings of 1‐2; please refer to Iyer et al [17] for further details [18]). Only the counselors’ voice recordings were used in this study. No information was provided that could be used to identify the callers or counselors for any of these calls.

### Ethical Considerations

This study was approved by the Swinburne Human Research Ethics Committee (Ref:20226835‐11907). The application was given a waiver of consent from the ethics committee for the use of nonidentifiable secondary research data. However, the research team also signed a confidentiality agreement that restricted the discussion of the contents of the recordings only within the research team. The secondary data and annotation results were saved on OneDrive for Business (Microsoft Corp) and were only accessible to the research team. No compensation was paid to participants in this study because they could not be identified by the research team.

### Annotation of Call Segments and Overall Call Empathy for Counselors

**Figure 1.**
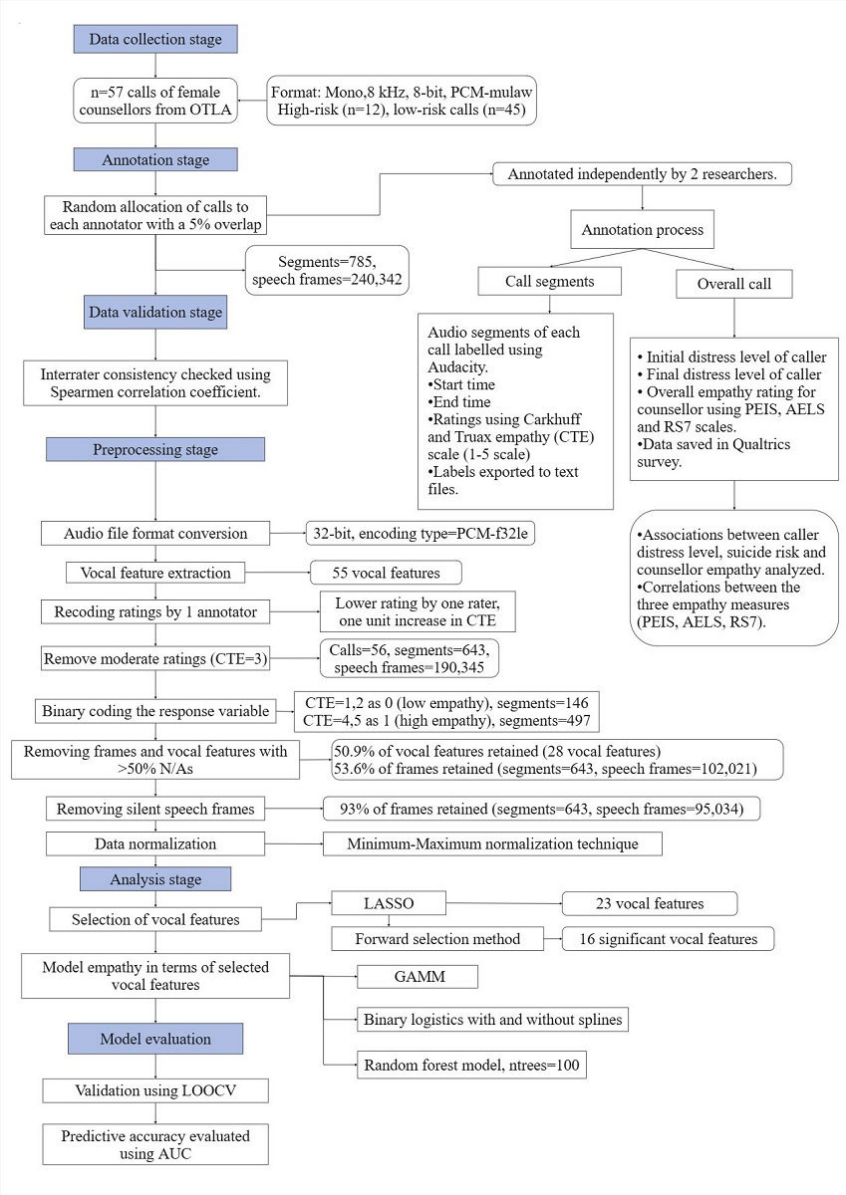
Overview of the voice analysis process for classifying empathy in counselor voices. AELS: Active-Empathic Listening Scale; AUC: area under the curve; CTE: Carkhuff and Truax Empathy; LASSO: Least Absolute Shrinkage and Selection Operator; LOOCV: Leave One Out Cross Validation; GAMM: generalized additive mixed model; N/A: not available; OTLA: On the Line Australia; PCM-f321e: pulse code modulation float; PEIS: Perceived Emotional Intelligence Scale; RS7: Rating Scale 7 (7-item).

Annotations of counselor empathy were conducted by 2 independent researchers (Inge Gnatt and Sarah Dunning) using RStudio (version 2024.04.2, build 764; R Foundation) [19]. The call annotators Inge Gnatt and Sarah Dunning were recruited via research team networks. Both had extensive experience working as counselors for a MH helpline service. Inge Gnatt had experience working as an annotator on a similar project. Segments of the counselor voices were selected from each call using Audacity (version 3.5.1, CMake Release Build; Muse Group & contributors) [20], ensuring that overlaps between the caller and responding counselor voices were minimized. Empathy displayed by the counselor within each call segment was rated using the Carkhuff and Truax Empathy (CTE) scale [21]. A weekly project team meeting, attended by a clinical psychologist (Maja Nedeljkovic), was used to reconcile any disparities in ratings. A Qualtrics web-based questionnaire was used to also collect data on the overall level of empathy displayed by the responding counselor during each call and to evaluate caller distress at the commencement and conclusion of each call. Figure 1 shows the flow of the voice analysis process during this study.

### Measures of Counselor Empathy and Caller Distress

The CTE scale was used to rate the audio segments selected from each call on a 5-point Likert-style scale (1=“low empathy” to 5=“high empathy”) [21]. Additionally, 3 measures were used to assess overall counselor empathy for each call. These 3 scales were modified to suit counselor-caller conversations through an iterative process in which members of the research team provided independent feedback to achieve the final questionnaire. Examples were developed by the annotators for each item included in these scales, ensuring clarity and consistency of ratings. The details for these scales are provided in Multimedia Appendix 1. The measurement scales included:

1. The Perceived Emotional Intelligence (PEI) Scale [22] identified variations in PEI in the counselor’s vocals. The PEI is a 20-item scale with each item scored with 1=“never or almost never true” and 7=“almost or almost always true.”
2. The Active-Empathic Listening (AEL) Scale [23] was modified appropriately to produce 10 items measuring empathic listening using a 7-point Likert-style scale with 1=“never or almost never true” and 7=“almost or almost always true.”
3. Rating Scale 7 (RS7): A single item 7-point Likert Scale [24] was used to rate overall empathy with 1=“low empathy” and 7=“high empathy.”

Finally, at the start and end of each call the annotators assessed the level of caller distress using the distress thermometer [25], a visual analogue 11-point scale (0=“no distress” to 10=“extreme distress”).

### Data Validation Through Interrater Consistency Check

Six (10%) calls were chosen at random to measure interrater reliability. The empathy ratings of the more experienced rater (Inge Gnatt) were used as the reference, against which the SD ratings were compared. Spearman correlation [26] was calculated for each of the 3 scales used to rate overall counselor empathy for each call. The Mann-Whitney *U* test was then used to check for the significance of differences between the ratings provided by the annotators.

### Relationships Between Perceptions of Empathy and Call Context

The associations between perceived counselor empathy and call context were explored using a combination of empathy ratings, caller distress at the beginning and end of the call, and caller suicide risk. Caller distress and suicide risk were correlated with perceived counselor empathy to evaluate the relationship between level of empathy and caller disposition.

### Preprocessing Stage: Audio File Format Conversion and Vocal Features Extraction

The input call recordings were obtained as 8 kHz sample rate, 8-bit depth .wav files. The encoding type of the files were transformed to PCM float format with 32-bit depth to ensure compatibility with RStudio for analysis. Vocal features (n=55) were extracted per 30-millisecond speech frames (50% overlap; Blackman windows) within each annotated segment using RStudio (version 2.7.0, *Soundgen* package [27]).

### Removing Moderate Ratings and Binary Coding Empathy Level

#### Overview

The vocal segments that scored a rating of 3 out of 5 on the CTE scale were removed (n=142, 18%) from further analysis because of their neutral empathic character. A binary response variable was then created for each of the 643 remaining segments (190,345 speech frames of 30 ms) with an empathy rating of 4‐5 coded as high empathy (n=146 segments) and a rating of 1‐2 coded as low empathy (n=497 segments).

#### Removing Missing Values in Speech Frames and Vocal Features

Vocal features and 30-millisecond speech frames with more than 50% missing values were removed to maintain the quality of data and improve the overall accuracy of the results. The resulting data retained 50.9% (n=28) of the original vocal features and 53.6% (n=102,021) of the original speech frames.

#### Removing Silent Speech Frames and Normalization

The silent speech frames were also removed from each of the 643 segments leaving 95,034 speech frames in the final analysis sample. Finally, the minimum and maximum normalizing technique [28] was applied to reduce the influence of background noise.

### Analysis Stage

#### Selection of Vocal Features

Variable selection was performed to identify vocal features that were strongly associated with empathy. L1 penalized LASSO (Least Absolute Shrinkage and Selection Operator) regression was used to select the most relevant variables by shrinking the coefficients of the least relevant variables to 0 [29]. Tenfold cross-validation was used to optimize the tuning parameter, lambda. Further refinement per the selected vocal features was then conducted using a forward stepwise regression model.

#### Models for Identifying Empathy Level With Selected Vocal Features

Three methods were used to classify low and high empathy segments based on this final set of selected vocal features. A generalized additive mixed model (GAMM) included vocal features as fixed effects, with each call treated as a random effect. Spline functions for the selected vocal features were used to account for nonlinearity [30]. The GAM function [31] of package *mgcv* [32] in RStudio was used for the analysis.

Random forest classification also allowed for nonlinear relationships using step functions while more efficiently processing large datasets [33]. This has been a prominent classification model used in studies involving vocal analysis [34,35]. The binary logistic regression model was the third model considered, again accounting for nonlinearity using splines, and was used in our study as the baseline model [36].

#### Model Evaluation

Probabilistic predictions for high versus low empathy levels were obtained for each segment using Leave One Caller Out Cross Validation. Based on these probabilities, receiver operating characteristic curves were created, and areas under the curves (AUCs) [37,38] were used to compare the reliability of these models. The Youden index [39] was used to decide the optimal cut point for classifying segments based on their estimated high versus low empathy probabilities.

## Results

### Overview

The following results were obtained from the 57 calls for female counselors. This sample of calls included 12 (12/57, 21%) calls at high risk of suicide and 45 (45/57,79%) calls at low risk of suicide.

### Annotation of Call Segments

Using the CTE scale, 146 (18.6%) segments showed low empathy, 142 (18.1%) showed medium empathy, and 497 (63.3%) segments demonstrated high empathy.

### Overall Call Empathy Ratings

Multimedia Appendix 2 provides descriptive statistics for the overall empathy ratings for the 57 calls using the 3 scales. Excellent reliability is observed from both the raters, Inge Gnatt and Sarah Dunning, for the PEI and AEL scales, with Cronbach α values above 0.9. The descriptive statistics and the Spearman correlation statistics between the annotators are shown in Multimedia Appendix 3. A strong agreement between raters was observed with the PEI measure and the RS7 empathy measure approaching statistical significance.

### Relationships Between Perceptions of Empathy and Call Context

The relationship of counselor empathy ratings with caller’s distress at the start and end of the call and suicide risk are shown in Table 1. While the correlations between the initial distress and the 3 empathy measures were not significant, a moderate, statistically significant negative correlation between the final distress of the caller and the empathy of the counselor was observed for both the PEI and RS7 (*P*<.01 and *P*<.001, respectively). The suicide risk of callers as measured using the C-SSRS, had a statistically significant but weak positive correlation with counselor empathy across the PEI and AEL measures. Strong statistically significant correlations among the 3 empathy measures were found, validating the empathy measurement process.

**Table 1.** Spearman correlation coefficients for caller distress and suicide risk with counselor empathy ratings^a^.

	Caller context			Ratings for counselor empathy		
Empathy rating	Initial distress	Final distress	Suicide risk	PEIS^b^	AELS^c^	RS7^d^
PEIS	–0.011	–0.414**	0.310*	1		
AELS	0.102	–0.233	0.308*	.822***	1	
RS7	–0.024	–0.443***	0.055	.847***	.851***	1

a ***P<.001 (2-tailed), **P<.01 (2-tailed), and *P<.05 (2-tailed).

b PEIS: Perceived Emotional Intelligence Scale.

c AELS: Active-Empathic Listening Scale.

d RS7: Rating Scale 7 (7-item).

### Analysis Stage

The selection of vocal features using LASSO to predict high versus low empathy used a cross-validation of the training dataset to reveal an optimum Log (Lambda) parameter=–8.542. The relationship between this parameter and the binomial deviance is shown in Multimedia Appendix 4. Using this lambda value, 23 vocal features were retained. These 23 vocal features were then passed on to the forward selection binary logistic regression model, which identified 16 significant vocal features as shown in Table 2.

**Table 2.** Results of the forward binary logistic regression model for vocal feature selection for identifying high versus low empathy in counselor voices.

Vocal features	Coefficient	*Z* value	*P* value
Depth of amplitude	–1.468	–15.994	<.001
Frequency of amplitude (Hz)	0.068	2.524	.01
Frequency of amplitude (Hz) via MS^a^	–0.462	–12.350	<.001
Purity of amplitude via MS	–0.834	–9.596	<.001
Amplitude (dB)	–3.391	–51.688	<.001
Dominant frequency (Hz)	0.985	3.136	.002
Entropy	3.47	20.843	<.001
Shannon entropy	–6.016	–25.27	<.001
Epoch	0.289	5.04	<.001
First formant frequency (Hz)	1.244	5.08	<.001
First formant width (Hz)	0.194	4.181	<.001
Second formant frequency (Hz)	–0.191	–2.808	.005
Second formant width (Hz)	–0.063	–1.802	.07
Third formant frequency (Hz)	–0.099	–1.521	.13
Third formant width (Hz)	0.004	0.118	.91
Spectral flux	0.11	1.435	.15
HNR^b^ (dB)	–0.37	2.869	.004
Spectral novelty	0.035	0.715	.47
Peak frequency (Hz)	–0.293	–1.827	.06
25th percentile frequency (Hz)	–1.311	–4.720	<.001
50th percentile frequency (Hz)	–0.262	–1.586	.11
Spectral centroid (Hz)	5.782	15.717	<.001
Spectral slope (Hz)	–3.944	–17.881	<.001

a MS: modulation spectrum.

b HNR: harmonics-to-noise ratio.

### Feature Extraction and Classification

**Figure 2.**
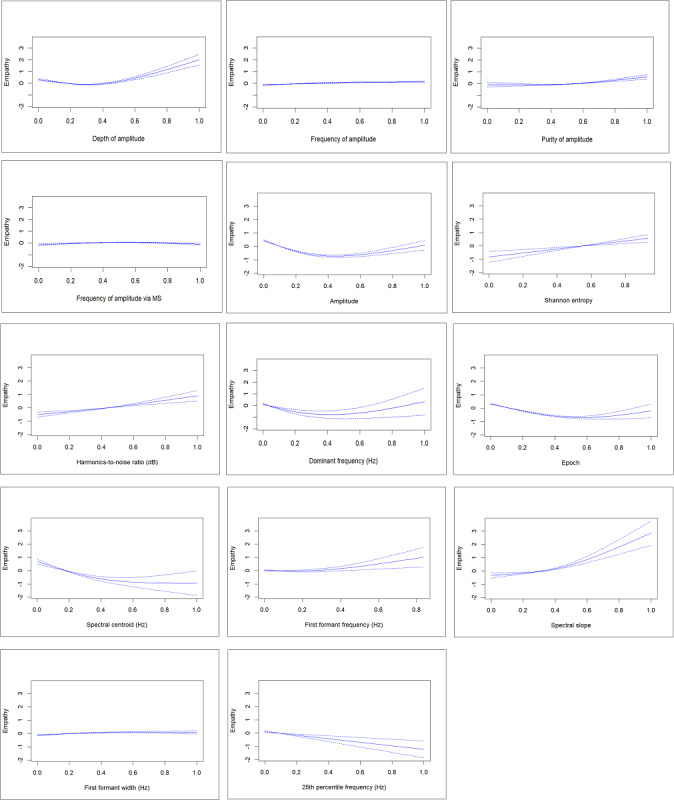
The smoothed relationship between the selected vocal features and the standardized predicted level of empathy displayed in counselor speech with 95% CIs. MS: modulation spectrum.

The results of the GAMM are shown in Multimedia Appendix 5. Of the 16 selected vocal features, the GAMM used only 14. Based on the effective *df* values, 2 of the vocal features (Shannon entropy and the 25th percentile frequency) show a linear relationship in the GAMM. Figure 2 illustrates the nonlinear nature of all other relationships. An AUC value of 0.605 was obtained for the GAMM [40].

The binary logistic classification model showed higher AUC values and nonoverlapping CIs at 95%. The DeLong test confirmed that the binary logistic model outperformed both the random forest (D=6.443, *df*=186283, *P*<.001) and GAMM (Z=5.846, *P*<.001) models [41]. However, this model achieved a lower classification accuracy than the other 2 methods, as shown in Table 3, suggesting that the probability cut point used for classification purposes was not ideal.

**Table 3.** Performance comparison of the 3 classification models for identifying high versus low counselor empathy.

Classification model	Accuracy (%)	AUC^a^ value	95% CIs
GAMM^b^	75	0.605	0.601‐0.609
Random forest	74	0.6	0.595‐0.604
Binary logistic regression	69	0.617	0.613‐0.622

a AUC: area under the curve.

b GAMM: generalized additive mixed model.

As illustrated in Figure 2, higher empathy was associated with higher values for the first formant frequency, dominant frequency, Shannon entropy, spectral slope, and harmonic-to-noise ratio. In contrast, higher empathy was associated with lower values for the 25th percentile frequency and spectral centroid. Finally, lower empathy was associated with intermediate values for depth of amplitude, amplitude, dominant frequency, epoch, and first formant frequency of speech.

The GAMM was able to differentiate between low and high 30-millisecond segments of speech to a classification accuracy of 75%. This was superior to both random forest and binary logistic regression models (74% and 69%, respectively).

Epoch (39%), amplitude (22%), and depth of amplitude (7%) were the top 3 vocal features contributing to empathic speech in the GAMM. The vocal features that contribute the most toward the identification of empathic speech vary across the 3 methods used, as shown in Multimedia Appendix 5.

For purposes of validation, the voice algorithm was also evaluated on a synthesized dataset of female voices created from a text-to-speech application. This approach yielded significant differences between high and low empathic voices in the validation dataset for the GAMM, random forest, and binary logistic regression classification models.

## Discussion

### Principal Findings

This study was undertaken to identify a range of vocal features that can predict the level of empathy exhibited in the recordings of female counselors and to accurately classify short segments of each recording according to low or high empathy ratings. We were successful in identifying 14 unique vocal features that significantly distinguished between low and high empathy ratings in the GAMM. Furthermore, we were able to successfully classify short segments of speech using these vocal features to an accuracy level of 75% using this model. Although this study considered only 57 calls, each call featured multiple segments annotated for the level of empathy, and each of these segments was further divided into 30-millisecond speech frames that were the observational points considered during the modeling process. Reducing the number of vocal features in the model led to lower AUC values, suggesting that the original model featuring 14 vocal features was not overfitted. Furthermore, the 95% AUC CIs for the binary logistic regression did not overlap those for the other 2 methods, confirming that the binary logistic regression model produced superior results.

Empathy is an important component of human interactions and social connections that promotes general well-being [42]. It is an essential component of MH care support, required to enhance therapeutic alliance and rapport building. Empathy embodies the ability to understand and compassionately reflect the range of feelings and experiences communicated by others. Empathic communication relies upon verbal (words and vocal features) and nonverbal means such as body language and facial expression [43]. However, it is only verbal expressions of empathy through vocal features that are the subject of this study.

The detection of empathy is traditionally based upon subjective human perception captured using standardized empathy scales or questionnaires [44]. Research on detecting empathic speech through voice feature extraction has been the focus of at least 2 recent studies, both showing similar accuracies to what we have found. For example, the first study by Chen et al [45] analyzed the acoustic prosodic features of speech recorded in YouTube (Google LLC) videos used for empathy training purposes. The use of these features resulted in classification accuracies of 59% (*F*_1_-score) when differentiating between empathy and neutral categories. A second study by Alam et al [46] explored acoustic and lexical features of empathic speech using annotations sourced from Italian call center conversations. In this study, classification using support vector machines yielded accuracies of 68.1%.

However, our study is unique in identifying a range of vocal features that identify the level of empathy using an ecologically valid dataset of counselor-caller conversations obtained from On the Line Australia, an Australian helpline service. Machine learning techniques are involved in both vocal feature selection and empathy classification. The 2 annotators chosen for empathy labeling purposes had a psychological background to strengthen the validity of this process. This led to the development of an algorithm that detects the human vocal features that are associated with empathic speech. This algorithm has the potential to enhance the training of counselors in the use of empathic speech and offers valuable insights into effective human communication in the MH care domain.

### Call Context and Counselor Empathy in a Crisis Helpline Setting

The analysis revealed that there is a strong negative correlation between the final distress level of the caller and the counselor’s level of empathy, suggesting that empathic communication with a caller can lower their level of distress by the end of the call. Higher levels of empathy allow the counselors to build rapport and trust with their patients, allowing for effective emotional support during a crisis. These findings align with the existing literature about the benefits of empathic interactions. The suicide risk of the caller was positively related to counselor empathy. This is an indication that counselors effectively recognize crisis situations, exhibiting higher levels of empathy when speaking to callers with high risks of suicide. It also confirms that the level of empathy displayed by counselors is adapted to the situation of the caller. These relationships of counselor empathy with caller distress and suicide risk confirm the importance of counselor empathy in the context of crisis helpline services.

### Characteristics of Empathic Vocals

This study has identified several vocal features associated with empathy. The depth of amplitude in speech reflects varying levels of loudness, emphasizing the expressiveness and dynamic nature of the human vocals apparent in empathic speech. A stable, more consistent emotional delivery during speech (purity of amplitude) also helps to convey empathy. Quieter vocals (amplitude and lower tonal frequency or dominant frequency) are also associated with higher empathy.

Higher first formant frequencies are associated with “a” vowel sound, which corresponds with high ratings of empathy. Additionally, a higher harmonic-to-noise ratio, indicating greater clarity and more pleasant-sounding vocals, is also associated with greater empathy. The spectral slope has a strong positive relationship to empathy, while the spectral centroid shows the opposite relationship. This indicates that a lower spectral centroid, with more low-frequency components, makes a speaker sound more empathic.

Based on these findings, it is evident that empathy in vocals is provided by a combination of multiple human vocal features, and variations in each of the features exert a different impact on empathy. In particular, the way that a specific threshold of loudness in the vocals decides the delivery of perceived empathy in the context of effective counseling provides compelling evidence. This further suggests that the right balance of each of these vocal features is needed, where stability, energy, and clarity play a pivotal role.

However, the study of empathy in vocals is a complex topic and has challenges. Especially the subjective nature of empathy perception is an area that requires further study. This study relied on empathy ratings provided by 2 psychology-trained female raters of English heritage. Different results may have been obtained if raters with different cultural, social, and educational backgrounds had been included [47].

### Vocal Feature Extraction and Empathy Classification

Three methods were used to study the association between empathy and relevant vocal features. A GAMM, a random forest, and a logistic regression approach with splines were fitted and compared using AUC values and using the Leave One Caller Out Cross Validation method for evaluating these classifiers. The accuracy of the empathy level classifications achieved was similar (75%, 74%, and 69%, respectively) when Youden index was used to choose the probability cut point, as were their AUC values (0.605, 0.600, and 0.617, respectively). The GAMM and binary logistic regression with splines required significantly more computational time compared to the random forest, which used 100 trees. Despite the significant improvement in AUC value with the logistic regression with splines in comparison to the random forest model, the AUC results do not seem to be dissimilar. However, the AUC 95% CIs for the binary logistic regression do not overlap with those for the other 2 methods, confirming that the logistic regression model provides a better fit to the data. This was further validated by the DeLong test results showing significant differences in AUC values of the binary logistic model compared to the other 2 methods. The partial overlap between the AUC CIs of the random forest and binary GAMMs indicates a significant difference in model fit. Therefore, the binary logistic classification model outperforms the other 2 methods in its ability to distinguish high-empathic speech from low-empathic speech. However, the slightly lower classification accuracy (69%) for this model suggests that the Youden method used to determine the probability cut point used for classification purposes may not be optimal.

These relatively low AUC values, also seen in other related research projects, can perhaps be partly attributed to the difficulties encountered in providing accurate ratings of empathy. The algorithms developed for detecting empathy from vocal features were reliant on the quality of the input data provided for the empathy ratings. A larger sample of raters and a larger sample of calls might have produced more reliable data, and this is recommended for future research in this area.

However, the multimodal approaches commonly used for empathy recognition through the use of words, vocals, visual signs, and psychological signals reflect the multifaceted nature of empathy [48]. The importance of facial expressions for recognizing empathy is particularly emphasized in this literature, where factors such as observation time and the type of emotion expressed significantly influence the accuracy of identification [49]. These multimodal approaches suggest that a higher accuracy in detecting empathy can be achieved when all these factors are collectively considered, rather than vocal approaches on their own.

### Limitations

The inherent differences of empathy perception among the annotators of this study were a concern in this study. An additional analysis was conducted to explore this further, incorporating a third annotator without psychological expertise and from a different cultural background (Multimedia Appendix 6). The findings from this analysis produced an even lower model performance, confirming that perceptions of empathy do vary between individuals and cultures. This analysis included both female and nonfemale counselor vocals, which may also have contributed to this poorer performance. While this finding underscores the complexity of recognizing empathy, it also highlights how cultural differences, personal experience, and psychological knowledge of individuals contribute to subjective perceptions. Therefore, future research would benefit from accommodating these differences in empathy perception within the model by including multiple annotators with varied backgrounds.

Another limitation of this study was due to the very small number of available male counselor recordings (n=13). The main analysis could therefore only be conducted on female vocals, limiting the generalization of our findings. Ideally it would have been possible to develop separate models for empathy in male and female counselors and to test whether there were significant differences between these models. Larger samples of counselor vocals would also have been preferable, providing more diversity in the data. Unfortunately, only a very small sample of male counselor calls was obtained. A recent study that focused on the vocal characteristics of distressed adults using machine learning techniques identified a significant difference between male and female vocal behaviors. This suggested that it would not be appropriate to use a single model to describe the voices of both male and female counselors [50]. For this reason, only female counselor voices were considered in this study. Recent research on machine learning applications has explored synthesized data as an avenue to address class imbalance, and this was considered as a possible remedy to address the lower representation of male voices in this study [51]. However, using synthetic speech, such as through text-to-speech applications, faces the challenge of reflecting the diversity inherent in natural human voices [52]. So, this option was not considered for boosting the number of male counselor calls, so as to make an analysis of these data possible.

However, this option was used when the algorithm developed in this study was validated on an external synthesized dataset consisting of female recordings generated using a text-to-speech application. These data also showed successful outcomes for the algorithm in differentiating between high and low empathic voices in female voices. These results highlight synthetic voice augmentation as a promising future research direction in machine learning applications. Alternatively, a balanced representation of gender, as well as cultural background, empathy levels, and individual characteristics, are necessary considerations for the counselor recordings used in future research of this nature.

A further limitation is the source of the calls used for this study. The context of a suicide helpline service is very specific, and it may be that more algorithmic success would have been possible in a less stressed environment. Additionally, for most of the calls, the level of empathy was assessed by a single rater. As mentioned above, it would have been preferable if a greater duplication of ratings could have been used to provide the dependent variable for the models that have been used to identify the level of empathy in counselor vocals. However, this pilot study, using machine learning techniques to identify vocal features related to empathy in female counselor voices, has shown promise. Therefore, further explorations of this approach with increases in the call sample size and a more balanced gender representation, and with more annotators to allow more duplication of ratings and more variation in annotator background while also including calls from more than 1 helpline service, will benefit future research on this topic.

### Implications of This Study

The importance of empathy in reducing the distress of callers confirms the need for the incorporation of empathic communication skills in training programs for counselors. Additionally, a statistically significant positive correlation was found between suicide risk and counselor empathy (*P*<.05). This suggests that counselors tend to be more empathic toward high-risk callers. This perhaps highlights the need for counselors to adhere to a more caller-centered approach, ensuring that empathy is consistently exhibited for both low-risk and high-risk callers. Resources should perhaps be allocated equally for all callers rather than having a crisis intervention strategy that is tailored to prioritize callers needing emergency support.

To the best of the authors’ knowledge, this is the first study that has identified the unique features of human vocals that are associated with the communication of empathy in a MH care setting. The results of this study have implications for the training of counselors and psychologists working for MH-related telephone helpline services. Additionally, these findings can serve as a training resource for MH professionals more broadly, enhancing the quality of care provided. The engineering of empathic chatbots, especially within a triage capacity, is another significant area of research that would benefit from the findings of this study [53].

However, collecting the vocal data of individuals for research purposes raises important ethical concerns. This research needs to prioritize user consent and caller privacy. It is recommended that people with lived experience in telephone counseling and MH be asked to assist with the co-design and coproduction of such research to ensure that any resulting training programs or monitoring systems are acceptable to users and meet consumer needs.

## Supplementary material

10.2196/67835 Multimedia Appendix 1 Online questionnaires used for data collection.

10.2196/67835 Multimedia Appendix 2 Descriptive statistics of the analysis.

10.2196/67835 Multimedia Appendix 3 Results of consistency between the raters.

10.2196/67835 Multimedia Appendix 4 The results of LASSO regression. LASSO: Least Absolute Shrinkage and Selection Operator.

10.2196/67835 Multimedia Appendix 5 Detailed results of the significance of the vocal features through GAMM, random forest, and binary logistics regression models. GAMM: Generalized Additive Mixed Model.

10.2196/67835 Multimedia Appendix 6 Impact of expanded dataset and rater diversity on voice analysis results.

10.2196/67835 Checklist 1 TRIPOD+AI (Transparent Reporting of a Multivariable Prediction Model for Individual Prognosis or Diagnosis+Artificial Intelligence) statement for reporting the modeling process.

## Abbreviations

AEL: Active-Empathic Listening
AUC: area under the curve
C-SSRS: Columbia Suicide Severity Rating Scale
CTE: Carkhuff and Truax Empathy
GAMM: generalized additive mixed model
LASSO: Least Absolute Shrinkage and Selection Operator
MH: mental health
PEI: Perceived Emotional Intelligence
RS7: Rating Scale 7
TRIPOD+AI: Transparent Reporting of a Multivariable Prediction Model for Individual Prognosis or Diagnosis+Artificial Intelligence
