# Supplementary material for: Machine Learning Approach to Identifying Empathy Using the Vocals of Mental Health Helpline Counselors: Algorithm Development and Validation
Source: JMIR Form Res. 2025 Apr 16;9:e67835. doi: 10.2196/67835 (PMC12017608; doi:10.2196/67835)
Supplement: Multimedia Appendix 1 [file formative-v9-e67835-s001.docx]

| 1. The PEI Scale | 1=Never or Almost never true (1) | 2 (2) | 3 (3) | 4 (4) | 5 (5) | 6 (6) | 7=Always or Almost always true (7) |
| --- | --- | --- | --- | --- | --- | --- | --- |
| **Perceiving Emotions (PE) - The ability to perceive emotions in oneself and others as well as in objects, art, stories, music, and other stimuli.** **Convey a sense that the counsellor listens openly to the caller’s emotions**  *e.g. - What is bringing you to call us today? What could be contributing to your distress right now?*  *Would you like to tell me what is going on for you to call us today?*  *Are you feeling better now?*  *What's causing this amount of distress and pain in your life right now? mmm, hmm *leaves space** (1) |  |  |  |  |  |  |  |
| **Convey a sense that the counsellor pays attention to the caller’s moods**  *e.g. - It sounds like you are very upset right now. It sounds like you've been feeling low. I can hear that you are feeling...mood-related* (2) |  |  |  |  |  |  |  |
| **Identify the caller’s emotions correctly**  *e.g. - I can understand your fear.  You sound very worried. Responds in a way that reflects the caller (i.e., congruent)* (3) |  |  |  |  |  |  |  |
| **Discern between different emotions**  *e.g. - I can understand you feeling sad about yourself after how he treated you and feeling disgusted about him. It sounds like you're feeling sad and lonely. It sounds like his actions have made you feel both sad and angry Asks - are you feeling? Responds to any changes to reflect the caller* (4) |  |  |  |  |  |  |  |
| **Tell apart the degree of emotions present (Identify the level of emotion present)**  *e.g. - I can understand that a lot has happened to you which is why you are in overwhelming pain. I can sense a deep sadness underneath the anger. Responds by matching e.g., slowing down, pace, tone of voice* (5) |  |  |  |  |  |  |  |
| **Using Emotions (UsE) - The ability to generate, use, and feel the emotion as necessary to communicate feelings or employ them in other cognitive processes.** **Convey a sense that the counsellor can feel what the caller is feeling**  *e.g. - I can feel that it's hard for you to go through this alone. It's so hard to feel this way. Response, through voice, other vocal prompters indicating they understand and are listening I can hear that you are feeling...emotion-related/your voice* (6) |  |  |  |  |  |  |  |
| **Convey a sense that the counsellor understands the caller’s point of view (POV)** *e.g. - It is fair that you are emotionally down, with the loss of your job. I can see what a hard situation this is for you. I can hear that you are feeling x, struggling with x Consider factual content.* (7) |  |  |  |  |  |  |  |
| **Respond in a way that makes the caller elicit difficult emotion.**  *e.g. - It is painful not having someone you could talk to. You must be feeling very alone. Dwells too long, not solution-focused, implies hopelessness* (8) |  |  |  |  |  |  |  |
| **Respond in a way that makes the caller elicit positive emotion.**  *e.g. - You are handling the situation well. You should be proud of yourself. That shows a lot of resilience, which is something to be proud of. Looks into strengths and connection* (9) |  |  |  |  |  |  |  |
| **Respond in a way that makes the caller feel that they are understood**  *e.g. - You've gone through a lot. Summarising* (10) |  |  |  |  |  |  |  |
| **Understanding Emotions (UnE) - The ability to understand emotional information, to understand how emotions combine and progress through relationship transitions, and to appreciate such emotional meanings.** **Convey a sense that the counsellor can be emotionally self-aware and insightful**  *e.g. - You are feeling worse because your family is not understanding your emotions. It sounds like being alone triggers uncomfortable feelings for you.* (11) |  |  |  |  |  |  |  |
| **Display some knowledge of complex emotions**  *e.g. -You are still in* ***grief*** *with the loss of your family. It must have been* ***terrifying.*** *I can hear that you are x, and there is a sense of y, there is a****struggle****.* (12) |  |  |  |  |  |  |  |
| **Respond empathetically to the caller**  *e.g. - I am glad you called today. You are welcome to call us again. I'm so glad you called. It's understandable, you are going through a lot I am glad you called Takes time to hear the person* (13) |  |  |  |  |  |  |  |
| **Describe/understand difficult emotions**  *e.g. - I feel like you may not have accepted the situation yet, and I am wondering if that is the source of your anger. Shame is a heavy weight to carry around. Clarifying function of naming emotions.* (14) |  |  |  |  |  |  |  |
| **Give the caller the impression that the counsellor is attempting to empathize**  *e.g. - I am sorry that you are feeling emotionally exhausted. That must be really difficult. Can I check that I am understanding the situation? How are you feeling?* (15) |  |  |  |  |  |  |  |
| **Managing Emotions (ME) - The ability to be open to feelings, and to modulate them in oneself and others so as to promote personal understanding and growth.** **Make decisions with the caller's feelings and thoughts.**  *e.g. - With your current situation, I suggest that you continue your weekly sessions with the psychologist. It sounds like you're ready to make a change. Would you like me to find the details for your local support service? Collaborative, problem solving* (16) |  |  |  |  |  |  |  |
| **Influence some of the caller’s thoughts**  *e.g. - May I suggest that it might be a good time to take a break and spend time taking care of yourself? You have good friends who want to support you. Shifting from catastrophising to more positive problem solving* (17) |  |  |  |  |  |  |  |
| **Provide psychologically minded advice**  *e.g. - I believe it helps if you think about this slowly, taking your own time. What's a small thing you could commit to doing every morning to help you to feel better? Helps them to understand what they are feeling and why* (18) |  |  |  |  |  |  |  |
| **Show some conscious thought before responding**  *e.g. - hmm, I agree with you. Space, not butting in* (19) |  |  |  |  |  |  |  |
| **Show openness to various emotions.**  *e.g. - Do you want to tell me something that makes you happy instead? Would you like to tell me why you feel this way? Tell me more about that?* (20) |  |  |  |  |  |  |  |

| 1. AELS Scale | 1=Never or Almost never true (1) | 2  (2) | 3  (3) | 4  (4) | 5  (5) | 6  (6) | 7=Always or  Almost always true (7) |
| --- | --- | --- | --- | --- | --- | --- | --- |
| **Sensing**  **The Counsellor is sensitive to what the caller is not saying.** *e.g. - Do you have something else you'd like to tell me? How are you feeling right now? Can you tell me a little more about... Can I ask a question/check in on....* (1) |  |  |  |  |  |  |  |
| **The Counsellor is aware of what the caller implies but does not say.** *e.g. - What did mean when you said that you might not be happy again? You must feel really betrayed. Sounds like you are going through a lot, there is a lot happening for you right now* (2) |  |  |  |  |  |  |  |
| **The Counsellor understand how the caller feels.** *e.g. - You have gone through something major and that is why you are feeling sad. I would feel angry too. Oh, that sounds really tough, I can understand why you might be feeling like that* (3) |  |  |  |  |  |  |  |
| **The Counsellor listen to more than just the spoken words.** *e.g. - You sound very tired. I can hear the stress in your voice. leaving space - hearing things in the background, asking about their current situation* (4) |  |  |  |  |  |  |  |
| **Processing**  **The Counsellor assure the caller that they will remember what the caller says.** *e.g. - You can tell me anything. I have a good memory. paraphrasing, checking, asking questions, staying present* (5) |  |  |  |  |  |  |  |
| **The Counsellor summarise points of agreement and disagreement when appropriate.** *e.g. - It is great that you agreed on seeing a counsellor on a regular basis. summarising, challenging gently where appropriate* (6) |  |  |  |  |  |  |  |
| **The Counsellor keep track of points the Caller make.** *e.g. - You mentioned earlier that you have been having these thoughts for a week now. You've had three major upsets in the past month. Staying focused* (7) |  |  |  |  |  |  |  |
| **Responding**  **The Counsellor assure the Caller that they are listening by using verbal acknowledgements.** *e.g. - hmmm, yeah, okay mmm hmm, ok, yes, go on, tell me more about..* (8) |  |  |  |  |  |  |  |
| **The Counsellor assures the Caller that they are receptive to their ideas.** *e.g. - Would you like to tell me more about that? That sounds like a good plan, can you tell me more about that? Ok, so help me understand* (9) |  |  |  |  |  |  |  |
| **The Counsellor asks questions that show their understanding of the Caller’s position.** *e.g. - It is alright for you to feel this way with everything that is going on for you in life. So, you're feeling angry right now and want them to leave you alone for a while, is that right? Can I check my understanding here? Can I ask a little more about x?* (10) |  |  |  |  |  |  |  |

| 1. Raters Scale (RS7) | 1=Low Empathy (1) | 2 (2) | 3 (3) | 4 (4) | 5 (5) | 6 (6) | 7=High Empathy (7) |
| --- | --- | --- | --- | --- | --- | --- | --- |
| The level of empathy observed in the Counsellor's voice from the perspective of the Rater (1) |  | | | | | | |
