# Supplementary material for: Machine Learning Approach to Identifying Empathy Using the Vocals of Mental Health Helpline Counselors: Algorithm Development and Validation
Source: JMIR Form Res. 2025 Apr 16;9:e67835. doi: 10.2196/67835 (PMC12017608; doi:10.2196/67835)
Supplement: Multimedia Appendix 2 [file formative-v9-e67835-s002.docx]

|  |  | Empathy Measures for Calls | | |
| --- | --- | --- | --- | --- |
|  | No. Calls | PEIS (/140) | AELS (/70) | RS7(/7) |
| Cronbach alpha (rater IG) | 16 | 0.968 | 0.971 | - |
| Cronbach alpha (rater SD) | 40 | 0.972 | 0.942 | - |
| Mean (SD) | 56 | 65.43(31.78) | 36.79(14.86) | 3.96(1.81) |
| Overall |  |  |  |  |
| Low Suicide Risk | 44 | 60.68 (31.61) | 35.05(14.39) | 3.95(1.85) |
| High Suicide Risk | 12 | 80.25 (31.51) | 45.17 (14.87) | 4.08(1.88) |
| Initial Distress Low (<=5) | 31 | 65.23(32.74) | 35.77(14.63) | 4.03(1.83) |
| Initial Distress High (>5) | 25 | 64.44(32.49) | 39.00(15.45) | 3.92(1.89) |
| Final Distress Low (<=5) | 41 | 71.02(32.98) | 37.95(15.88) | 4.27(1.87) |
| Final Distress High (>5) | 15 | 48.07(21.76) | 35.20(12.30) | 3.2(1.57) |

**Table S1.** Descriptive statistics for empathy measures based on overall ratings.

PEIS = The Perceived Emotional Intelligence Scale, AELS = The Active-Empathic Listening Scale; RS7 = Rating Scale 7 (7-item)
