# Supplementary material for: Machine Learning Approach to Identifying Empathy Using the Vocals of Mental Health Helpline Counselors: Algorithm Development and Validation
Source: JMIR Form Res. 2025 Apr 16;9:e67835. doi: 10.2196/67835 (PMC12017608; doi:10.2196/67835)
Supplement: Multimedia Appendix 3 [file formative-v9-e67835-s003.docx]

| Rater Pair | Empathy Measure | Spearman’s ρ (rho) | P-value | Rater IG  Mean (SD) | Rater SD  Mean (SD) |
| --- | --- | --- | --- | --- | --- |
| IG and SD | PEIS | 0.83 | 0.058 | 93.46(34.08) | 94.22(23.51) |
| IG and SD | AELS | 0.43 | 0.419 | 44.75(17.40) | 42.60(12.82) |
| IG and SD | RS7 | 0.79 | 0.060 | 5.00(2.16) | 6.05(1.52) |

**Table S1.** Spearman correlation between annotators' empathy scores.

PEIS = The Perceived Emotional Intelligence Scale, AELS = The Active-Empathic Listening Scale; RS7 = Rating Scale 7 (7-item)

Note: Ratings by SD were recalibrated with the addition of one unit to all items based on the ratings of the more experienced rater (IG).
