# Supplementary figures and images for: Machine Learning Approach to Identifying Empathy Using the Vocals of Mental Health Helpline Counselors: Algorithm Development and Validation
Source: JMIR Form Res. 2025 Apr 16;9:e67835. doi: 10.2196/67835 (PMC12017608; doi:10.2196/67835)

**Figure S1.** Plot of log lambda versus binomial deviance
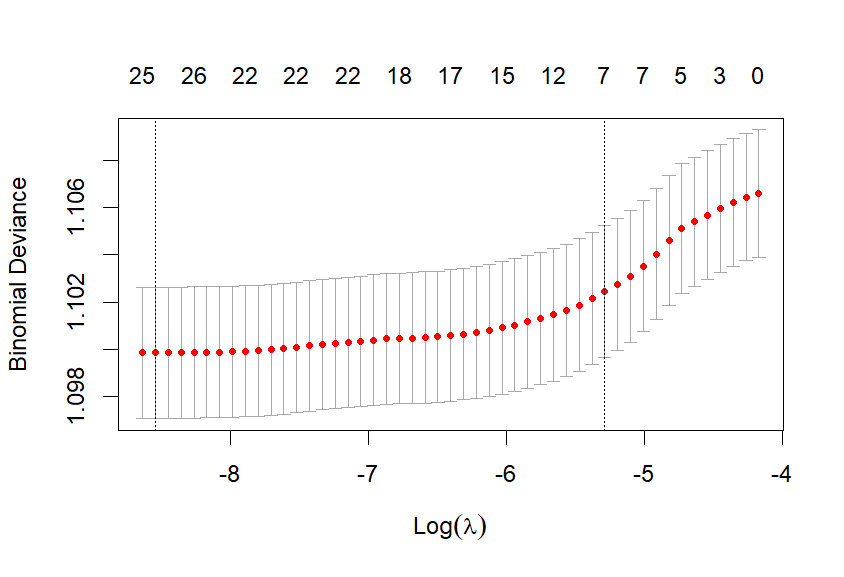
.

Supplement: Multimedia Appendix 4 [file formative-v9-e67835-s004.docx]
