# Supplementary material for: Machine Learning Approach to Identifying Empathy Using the Vocals of Mental Health Helpline Counselors: Algorithm Development and Validation
Source: JMIR Form Res. 2025 Apr 16;9:e67835. doi: 10.2196/67835 (PMC12017608; doi:10.2196/67835)
Supplement: Multimedia Appendix 5 [file formative-v9-e67835-s005.docx]

| Vocal features | GAMM (F-values) | Random Forest (Importance) | Binary Logistic (Chi-squared) |
| --- | --- | --- | --- |
| Epoch | 282.152 | 63.990 | 221.157 |
| Amplitude (dB) | 159.958 | 121.790 | 2790.709 |
| Depth of amplitude (0 or 1) | 52.912 | 41.835 | 193.369 |
| Frequency of amplitude (Hz) | 39.905 | 45.473 | 59.816 |
| HNR (dB) | 34.299 | 40.135 | 54.270 |
| Spectral centroid (Hz) | 31.245 | 42.361 | 276.214 |
| Spectral slope (Hz) | 26.693 | 46.001 | 255.073 |
| Purity of amplitude via MS | 22.066 | 62.328 | 32.755 |
| Dominant frequency (Hz) | 17.947 | 65.859 | 6.157 |
| Shannon entropy | 15.927 | 38.685 | 289.265 |
| 25^th^ percentile frequency (Hz) | 14.844 | 17.954 | 18.015 |
| First formant width (Hz) | 12.324 | 44.987 | 46.597 |
| Frequency of amplitude via MS (Hz) | 9.847 | 33.229 | 96.896 |
| First formant frequency (Hz) | 3.943 | 35.793 | 28.175 |
| Second formant frequency (Hz) | 2.969 | 44.984 | 30.790 |
| Entropy | 0.403 | 42.167 | 91.270 |

Based on the results in Table S1, it is evident that using different methods to identify how significance of vocal features of empathic voice gives different values.

**Table S1.** Comparison of vocal feature significance across GAMM, random forest, and binary logistics regression models.

Hz = Hertz, dB = Decibels

**Table S2.** Approximate significance of splines in GAMM.

| Vocal features (Smooth terms) | Effective df | F-value | P-value |
| --- | --- | --- | --- |
| Epoch | 1.976 | 282.152 | <.001 |
| Amplitude (dB) | 1.993 | 159.958 | <.001 |
| Depth of amplitude (0 or 1) | 1.990 | 52.912 | <.001 |
| Frequency of amplitude (Hz) | 1.843 | 39.905 | <.001 |
| HNR (dB) | 1.409 | 34.299 | <.001 |
| Spectral centroid (Hz) | 1.925 | 31.245 | <.001 |
| Spectral slope (Hz) | 1.933 | 26.693 | <.001 |
| Purity of amplitude via MS | 1.914 | 22.066 | <.001 |
| Dominant frequency (Hz) | 1.947 | 17.947 | <.001 |
| Shannon entropy | 1.006 | 15.927 | <.001 |
| 25^th^ percentile frequency (Hz) | 1.002 | 14.844 | <.001 |
| First formant width (Hz) | 1.867 | 12.324 | <.001 |
| Frequency of amplitude via MS (Hz) | 1.947 | 9.847 | <.001 |
| First formant frequency (Hz) | 1.838 | 3.943 | 0.037 |
| Second formant frequency (Hz) | 1.025 | 2.969 | 0.081. |
| Entropy | 1.008 | 0.403 | 0.526 |

df= Degrees of freedom, Hz = Hertz, dB = Decibels

**Table S3.** The importance of vocal features when using the random forest classification with training data.

| Vocal features | %IncMSE | IncNodePurity |
| --- | --- | --- |
| Amplitude | 121.790 | 1282.333 |
| Dominant frequency (Hz) | 65.859 | 911.954 |
| Epoch | 63.990 | 864.479 |
| Purity of amplitude via MS | 62.328 | 742.716 |
| Spectral slope (Hz) | 46.001 | 589.99 |
| Frequency of amplitude (Hz) | 45.473 | 826.174 |
| First formant width (Hz) | 44.987 | 593.126 |
| Second formant frequency (Hz) | 44.984 | 619.989 |
| Spectral centroid (Hz) | 42.361 | 573.875 |
| Entropy | 42.167 | 580.505 |
| Depth of amplitude (0 or 1) | 41.835 | 815.796 |
| HNR (dB) | 40.135 | 604.399 |
| Shannon entropy | 38.685 | 577.294 |
| First formant frequency (Hz) | 35.793 | 614.511 |
| Frequency of amplitude via MS (Hz) | 33.229 | 689.893 |
| 25^th^ percentile frequency (Hz) | 17.954 | 307.636 |

%IncMSE - Percent Increase in Mean Squared Error

IncNodePurity - Increase in Node Purity

Hz = Hertz, dB = Decibels

Amplitude and Dominant frequency show higher values of percentage IncMSE and IncNodePurity ensuring the significance of these features in predicting empathy in the model compared to other vocal features.

**Table S4.** The results of binary logistics regression with training data.

| Vocal features | Effective df | Reference df | Chi-squared value | P-value |
| --- | --- | --- | --- | --- |
| Depth of amplitude (0 or 1) | 1.987 | 2.000 | 193.369 | <.001 |
| Frequency of amplitude (Hz) | 1.979 | 2.000 | 59.816 | <.001 |
| Frequency of amplitude via MS (Hz) | 1.956 | 1.998 | 96.896 | <.001 |
| Purity of amplitude via MS | 1.921 | 1.994 | 32.755 | <.001 |
| Amplitude | 1.997 | 2.000 | 2790.709 | <.001 |
| Dominant frequency (Hz) | 1.576 | 1.818 | 6.157 | 0.095 |
| Entropy | 1.866 | 1.980 | 91.270 | <.001 |
| Shannon entropy | 1.677 | 1.893 | 289.265 | <.001 |
| Epoch | 1.993 | 2.000 | 221.157 | <.001 |
| First formant frequency (Hz) | 1.870 | 1.982 | 28.175 | <.001 |
| First formant width (Hz) | 1.967 | 1.999 | 46.597 | <.001 |
| Second formant frequency (Hz) | 1.943 | 1.996 | 30.790 | <.001 |
| HNR | 1.984 | 1.999 | 54.270 | <.001 |
| 25^th^ percentile frequency (Hz) | 1.993 | 1.999 | 18.015 | <.001 |
| Spectral centroid (Hz) | 1.937 | 1.994 | 276.214 | <.001 |
| Spectral slope (Hz) | 1.746 | 1.933 | 255.073 | <.001 |

df= Degrees of freedom, Hz = Hertz, dB = Decibels

All the vocal features are significant based on the results shown in Table S4 except dominant frequency in voice.
