# Supplementary material for: Machine Learning Approach to Identifying Empathy Using the Vocals of Mental Health Helpline Counselors: Algorithm Development and Validation
Source: JMIR Form Res. 2025 Apr 16;9:e67835. doi: 10.2196/67835 (PMC12017608; doi:10.2196/67835)
Supplement: Multimedia Appendix 6 [file formative-v9-e67835-s006.docx]

Additional analysis results

The impact of additional factors such as including male counsellor voices in the sample while an additional annotator without a psychological background and coming from a different cultural standpoint were considered during this additional analysis. A sample of n=120 calls (27 of high risk, n=93 of low risk) were used with n=50 calls allocated to the third rater. Out of the 120 calls, n=95 was of female counsellors while the rest (n=25) is of male counsellors.

| Vocal features | GAMM (F-values) | Random Forest (Importance) | Binary Logistic (Chi-squared) |
| --- | --- | --- | --- |
| Epoch | 371.278 | 93.774 | 2989.487 |
| 25^th^ percentile frequency (Hz) | 78.82 | 34.21 | 52.799 |
| Frequency of amplitude (Hz) | 71.946 | 61.961 | 84.897 |
| Amplitude (dB) | 29.772 | 102.972 | 1645.790 |
| Dominant frequency (Hz) | 26.730 | 71.480 | 35.266 |
| First formant width (Hz) | 22.086 | 43.996 | 77.361 |
| Entropy | 5.938 | 65.078 | 81.323 |
| Second formant width (Hz) | 4.959 | 25.60 | 26.830 |
| Spectral novelty | 4.678 | 9.589 | 6.046 |
| Depth of amplitude (0 or 1) | 4.396 | 50.948 | 220.121 |
| 75^th^ percentile frequency (Hz) | 3.566 | 47.460 | 17.782 |
| Third formant width (Hz) | 0.439 | 22.487 | 2.592 |

**Table S1.** Comparison of vocal feature significance across GAMM, random forest, and binary logistics regression models in the additional analysis.

| Variable | Estimate | SE | z value | P-value |
| --- | --- | --- | --- | --- |
| Depth of amplitude (0 or 1) | -1.112 | 0.079 | -14.091 | < 0.000 |
| Frequency of amplitude (Hz) | 0.111 | 0.022 | 4.962 | 0.000 |
| Amplitude (dB) | -2.159 | 0.062 | -34.770 | < 0.000 |
| Dominant frequency (Hz) | 0.612 | 0.251 | 2.433 | 0.015 |
| Entropy | -0.227 | 0.087 | -2.607 | 0.009 |
| Epoch | 2.974 | 0.059 | 50.588 | < 0.000 |
| First formant width (Hz) | 0.248 | 0.035 | 7.077 | 0.000 |
| Second formant width (Hz) | 0.127 | 0.028 | 4.490 | 0.000 |
| Third formant width (Hz) | 0.095 | 0.029 | 3.269 | 0.001 |
| Harmonics-to-noise ratio -HNR (dB) | 0.029 | 0.113 | 0.256 | 0.798 |
| Spectral novelty | -0.109 | 0.041 | -2.637 | 0.008 |
| Peak frequency (Hz) | 0.127 | 0.126 | 1.011 | 0.312 |
| 25th percentile frequency (Hz) | 0.385 | 0.187 | 2.053 | 0.040 |
| 50th percentile frequency (Hz) | 0.193 | 0.103 | 1.867 | 0.062 |
| 75th percentile frequency (Hz) | -0.263 | 0.059 | -4.500 | 0.000 |
| Roughness | -0.071 | 0.074 | -0.964 | 0.335 |
| Spectral slope (Hz) | -0.024 | 0.095 | -0.255 | 0.799 |

**Table S2.** Results of for variable selection of the additional analysis.

SE = standard error, Hz = Hertz, dB = Decibels

**Table S3.** Approximate significance of splines for GAMM.

| Vocal features (Smooth terms) | Effective df | Reference. df | F-value | P-value |
| --- | --- | --- | --- | --- |
| Depth of amplitude (0 or 1) | 1.903 | 1.903 | 4.396 | 0.014 |
| Frequency of amplitude (Hz) | 1.005 | 1.005 | 71.946 | < 0.000 |
| Amplitude (dB) | 1.982 | 1.982 | 29.772 | < 0.000 |
| Dominant frequency (Hz) | 1.917 | 1.917 | 26.730 | < 0.000 |
| Entropy | 1.006 | 1.006 | 5.938 | 0.015 |
| Epoch | 1.997 | 1.997 | 371.278 | < 0.000 |
| First formant width (Hz) | 1.944 | 1.944 | 22.086 | < 0.000 |
| Second formant width (Hz) | 1.204 | 1.204 | 4.959 | 0.017 |
| Third formant width (Hz) | 1.291 | 1.291 | 0.439 | 0.698 |
| Spectral novelty | 1.759 | 1.759 | 4.678 | 0.007 |
| 25^th^ percentile frequency (Hz) | 1.992 | 1.992 | 78.82 | < 0.000 |
| 75^th^ percentile frequency (Hz) | 1.822 | 1.822 | 3.566 | 0.088 |

df = degrees of freedom, Hz = Hertz, dB = Decibels

**Table S4.** Performance metrics for classification models.

| Classification method | AUC | 95% CI (AUC) | Accuracy percentage |
| --- | --- | --- | --- |
| Random Forest | 0.536 | 0.533-0.539 | 54.11% |
| Binary Logistics regression with splines | 0.565 | 0.562-0.569 | 55.23% |

CI= Confidence Intervals, AUC = Area under the curve

**Table S5.** The importance of vocal features when using the random forest classification with training data.

| Vocal features | %IncMSE | IncNodePurity |
| --- | --- | --- |
| Amplitude (dB) | 102.972 | 1742.605 |
| Epoch | 93.774 | 1957.971 |
| Dominant frequency (Hz) | 71.480 | 1495.309 |
| Entropy | 65.078 | 1191.750 |
| Frequency of amplitude (Hz) | 61.961 | 1490.508 |
| Depth of amplitude (0 or 1) | 50.948 | 1459.642 |
| 75^th^ percentile frequency (Hz) | 47.460 | 947.046 |
| First formant width (Hz) | 43.996 | 1150.787 |
| 25^th^ percentile frequency (Hz) | 34.21 | 685.426 |
| Second formant width (Hz) | 25.60 | 1044.258 |
| Third formant width (Hz) | 22.487 | 1001.806 |
| Spectral novelty | 9.589 | 952.283 |

%IncMSE - Percent Increase in Mean Squared Error

IncNodePurity - Increase in Node Purity

Hz = Hertz, dB = Decibels

Amplitude and Epoch show higher values of percentage IncMSE and IncNodePurity ensuring the significance of these features in predicting empathy in the model compared to other vocal features.

**Table S6.** The results of binary logistics regression with training data.

| Vocal features | Effective df | Reference df | Chi-squared value | P-value |
| --- | --- | --- | --- | --- |
| Depth of amplitude (0 or 1) | 1.984 | 2.000 | 220.121 | < 0.000 |
| Frequency of amplitude (Hz) | 1.981 | 2.000 | 84.897 | < 0.000 |
| Amplitude (dB) | 1.997 | 2.000 | 1645.790 | < 0.000 |
| Dominant frequency (Hz) | 1.965 | 1.999 | 35.266 | < 0.000 |
| Entropy | 1.983 | 2.000 | 81.323 | < 0.000 |
| Epoch | 1.994 | 2.000 | 2989.487 | < 0.000 |
| First formant width (Hz) | 1.912 | 1.992 | 77.361 | < 0.000 |
| Second formant width (Hz) | 1.569 | 1.814 | 26.830 | < 0.000 |
| Third formant width (Hz) | 1.021 | 1.041 | 2.592 | 0.116 |
| Spectral novelty | 1.004 | 1.007 | 6.046 | 0.014 * |
| 25^th^ percentile frequency (Hz) | 1.002 | 1.005 | 52.799 | < 0.000 |
| 75^th^ percentile frequency (Hz) | 1.922 | 1.994 | 17.782 | 0.000 |

df = degrees of freedom, Hz = Hertz, dB = Decibels

All the vocal features are significant based on the results shown in Table S6 except dominant Third formant width in voice.
